# Pubiotomy, Cæsarean Section, and Induction

**Published:** 1915-10-16

**Authors:** 


### PUBIOTOMY, CÆSAREAN SECTION, AND INDUCTION.

Professor Whitridge Williams, of Baltimore, is well known as an advocate of pubiotomy, an operation for delivery of women with contracted pelvis which is not much favoured in this country. In a recent paper read before the Buffalo Academy of Medicine he defines thoroughly his present position and teaching. His experience has taught him greater conservatism in the employment of pubiotomy than he formerly practised; except in funnel pelves in young women he does not now regard it as an elective operation. In contraction of the bones alone his ideal is to differentiate between those who will require Cæsarean section at the onset of labour, and those in whom there is a fair chance of spontaneous delivery. Pubiotomy can be held in reserve for the latter when failure of the head to engage after a prolonged second stage demonstrates that the prognosis was erroneous. Pubiotomy does not, he holds, compete with elective Cæsarean section at the onset of labour, but is much safer than conservative Cæsarean section late in the second stage, especially if the patient has been handled by anyone whose technique is questionable. What with pubiotomy and Cæsarean section, employed on Professor Williams' lines, he believes the induction of premature labour is an operation which can be definitely abandoned. A colleague of Professor Williams, Dr. H. K. Thoms, has published, in the same issue of the *American Journal of Obstetrics*, a research upon the funnel pelvis, which he finds much commoner than has usually been supposed. The modified Sims posture increases temporarily the contracted antero-posterior diameter of the outlet in these cases very excellently; but for severe degrees of the deformity he recommends pubiotomy, which often transforms a bad funnel pelvis into one with almost normal diameters.

